# The Critical Clock: Time-Dependent Inflammation, Catabolism and Immunosuppression Syndrome Is Associated with ICU Mortality

**DOI:** 10.3390/diagnostics16193254

**Published:** 2026-10-08

**Authors:** Valery Likhvantsev, Levan Berikashvili, Petr Polyakov, Mikhail Yadgarov, Artem Kuzovlev, Alexey Yakovlev, Andrey Grechko

**Affiliations:** 1 Department of Clinical Trials, Federal Research and Clinical Center of Intensive Care Medicine and Rehabilitology, 107031 Moscow, Russia; lberikashvili@fnkcrr.ru (L.B.); p.polyakov@fnkcrr.ru (P.P.); myadgarov@fnkcrr.ru (M.Y.); artem_kuzovlev@fnkcrr.ru (A.K.); ayakovlev@fnkcrr.ru (A.Y.); fnkcrr@fnkcrr.ru (A.G.); 2 Department of Anesthesiology and Intensive Care, I.M. Sechenov First Moscow State Medical University of the Russian Ministry of Health, 119048 Moscow, Russia

**Keywords:** chronic critical illness, inflammation, catabolism, immunosuppression syndrome, time-dependent exposure, mortality, PICS, RICD

## Abstract

**Background/Objectives**: Chronic critical illness (CCI) is increasingly recognized as a major challenge in intensive care, yet its definition remains heterogeneous. The triad of inflammation, catabolism, and immunosuppression (ICS) is central to proposed CCI pathophysiology, but the association between its changing components and prognosis has not been systematically evaluated. We examined whether the current presence of the biomarker-defined ICS syndrome was associated with ICU mortality. **Methods**: In this retrospective single-center cohort study, we analyzed 820 ICU patients from the Russian Intensive Care Dataset (RICD v3.0) who had at least one episode of the biomarker-defined ICS syndrome, operationally defined by concurrent C-reactive protein > 20 mg/L, albumin < 30 g/L, and absolute lymphocyte count < 0.8 × 10^9^/L within a 24 h period. After the index ICS syndrome event, each patient’s ICU course was classified according to the current syndrome status: ICS syndrome present (all three criteria met) or ICS syndrome absent (at least one criterion not met). We compared organ dysfunction, laboratory parameters, life-support interventions, and mortality between periods with the syndrome present and absent using linear mixed-effects models, generalized estimating equations, and time-dependent Cox regression. Mortality density was calculated per 1000 patient-days. **Results**: Periods with the ICS syndrome present accounted for 5271 (22.2%) of 23,796 post-index patient-days and periods with the syndrome absent accounted for 18,525 (77.8%). Variables not used to define the syndrome showed greater contemporaneous severity while the ICS syndrome was present, including higher SOFA and SOFA-2 scores, worse neurological status, lower platelet and total protein levels, and more frequent mechanical ventilation initiation (32.8% vs. 23.6%; OR: 1.59, *p* < 0.001). The ICS syndrome was present at the time of 133 of 243 deaths (54.7%), with a mortality density of 25.23 versus 5.94 per 1000 patient-days (IRR: 4.25, 95% CI: 3.30–5.47). In time-dependent Cox regression, the current presence versus absence of the biomarker-defined ICS syndrome was associated with a higher hazard of ICU death (unadjusted HR: 4.02, 95% CI: 2.14–7.55; adjusted HR: 3.86, 95% CI: 1.86–8.02). **Conclusions**: In this cohort, the current presence of the biomarker-defined ICS syndrome was associated with substantially higher ICU mortality risk than its absence. These findings support dynamic risk assessment and closer clinical evaluation; however, the biomarker combination may partly reflect contemporaneous illness severity, and the present analysis does not establish a causal effect of syndrome presence or an independent effect of cumulative prior time with the syndrome present.

## 1. Introduction

Chronic critical illness (CCI) has emerged as a substantial and growing challenge in contemporary healthcare systems, imposing a significant burden on intensive care unit (ICU) resources and healthcare expenditures worldwide [1]. Epidemiological evidence indicates that approximately 7.6% of ICU admissions progress to a protracted course, characterized by prolonged dependence on organ support and heightened risk of adverse outcomes [2]. Despite its clinical relevance, no universally accepted definition of CCI currently exists. Historically, diagnostic criteria have relied on arbitrary temporal thresholds such as minimum ICU length of stay, duration of mechanical ventilation, or the presence of a tracheostomy [3,4,5]. However, this approach has resulted in substantial heterogeneity in reported prevalence and mortality, depending on the specific thresholds applied [6]. Such variability precludes meaningful comparisons across studies and hampers the development of standardized prevention and treatment protocols.

In response to these limitations, recent investigations have shifted focus towards identifying the underlying pathophysiological mechanisms that accompany the transition from acute to chronic critical illness. A predominant concept is the triad of persistent inflammation, catabolism, and immunosuppression, commonly termed PICS (persistent inflammation, catabolism, and immunosuppression syndrome) [7]. For the purposes of the present study, we use the term biomarker-defined ICS syndrome to denote the contemporaneous laboratory pattern without a prespecified duration. It is operationally defined by concurrent fulfillment of three criteria: C-reactive protein > 20 mg/L, serum albumin < 30 g/L, and absolute lymphocyte count < 0.8 × 10^9^/L. This designation is analytical rather than diagnostic, and PICS is reserved for the persistent syndrome in which a temporal component is explicit.

Conventional PICS formulations commonly incorporate a time criterion, such as an ICU stay exceeding 14 days [8,9]. In our earlier work, concurrent fulfillment of the three ICS biomarker criteria at any point during the ICU course, irrespective of ICU length of stay, was associated with increased mortality. This observation motivated evaluation of the biomarker-defined ICS syndrome independently of an arbitrary time threshold; however, it does not establish these criteria as a universally accepted replacement for conventional CCI definitions.

Building upon these observations, we have proposed that the three components of ICS may not evolve synchronously. Consequently, the biomarker-defined ICS syndrome may be present during some intervals and absent during others. In this study, ‘ICS syndrome absent’ means only that at least one of the three laboratory criteria is not fulfilled. It does not imply resolution of CCI, clinical recovery, or that a preceding period with the syndrome present is unrelated to a subsequent outcome. This temporal pattern has not been previously examined in relation to clinical outcomes. It remains unknown whether mortality risk differs according to the current presence versus absence of the biomarker-defined ICS syndrome within the same patient.

Current therapeutic strategies for CCI have not shown consistent efficacy, and the temporal heterogeneity of the ICS syndrome may be clinically relevant. Characterizing the association between the current presence versus absence of the biomarker-defined ICS syndrome and mortality may support dynamic risk assessment and generate hypotheses for prospective phase-specific studies; an observational comparison of syndrome status cannot, by itself, establish that changing the status will improve outcome.

Accordingly, the objective was to compare the contemporaneous risk of ICU death during periods with the biomarker-defined ICS syndrome present versus absent after the index syndrome event.

## 2. Materials and Methods

### 2.1. Source of Data

This study was based on data from the Russian Intensive Care Dataset (RICD) v3.0, an institutional de-identified database maintained by the Federal Research and Clinical Center of Intensive Care Medicine and Rehabilitology (FRCC ICMR).

FRCC ICMR is a tertiary medical center focused on the treatment and investigation of prolonged critical illness (PCI) and chronic critical illness (CCI). The database includes medical records of patients treated at FRCC ICMR between December 2017 and September 2025. The dataset is structured as 11 linked relational tables and contains 10,938 hospital admissions from 8135 patients, including 4527 ICU admissions and 4050 patients with at least one ICU stay.

### 2.2. Study Design and Setting

This retrospective single-center cohort study was conducted using real-world clinical data from the RICD v3.0. It included ICU patients with at least one biomarker-defined ICS syndrome event and compared subsequent periods with the syndrome present and absent as mutually exclusive categories of a time-dependent exposure.

For the purpose of this study, we screened all ICU admissions. Patients were eligible if they met the following inclusion criteria: (1) first ICU admission, with readmissions excluded, (2) age 18 years or older, and (3) at least one index episode of the biomarker-defined ICS syndrome, operationally defined as the simultaneous presence within a 24 h period of C-reactive protein > 20 mg/L, albumin < 30 g/L, and an absolute lymphocyte count < 0.8 × 10^9^/L. Exclusion criteria were as follows: (1) ICU length of stay < 24 h and (2) absence of documented ICD-10 diagnosis records. The ICD-10 criterion was a prespecified data-quality requirement intended to exclude admissions lacking admission-level clinical information needed to characterize case mix; ICD coding was not part of the exposure or outcome definition. The first episode meeting all three ICS syndrome criteria served as an operational index event for longitudinal observation and was not intended to replace conventional time- or organ-support-based definitions of CCI.

All eligible cases from the dataset were included without formal sample size calculation. The local Ethics Committee confirmed that no formal approval was required for this study due to the de-identified nature of the data (Committee decision No. 4/23/2 dated 20 December 2023). The study was conducted in accordance with the Strengthening the Reporting of Observational Studies in Epidemiology (STROBE) statement [10]. The STROBE checklist is provided in Supplementary S1.

### 2.3. Data Management

The extracted variables included demographic and anthropometric characteristics, admission diagnoses, ICU admission status, clinical severity scores, laboratory parameters, life-support variables, and ICU outcomes.

Clinical severity was assessed using GCS, FOUR, SOFA, and SOFA-2 scores, including individual SOFA and SOFA-2 organ-domain scores. SOFA-2 is the 2025 revision of the Sequential Organ Failure Assessment score; it retains six organ systems and a 0–24 point range while updating thresholds and contemporary organ-support modalities [11]. Laboratory variables included complete blood count parameters, creatinine, urea, arterial lactate, AST, ALT, ALP, total protein, albumin, CRP, and absolute lymphocyte and neutrophil counts. Life-support variables included initiation of invasive mechanical ventilation (MV), any vasoactive-inotropic support, and VIS. For the period-specific analysis, MV initiation was defined as the first documented start of invasive MV occurring after the index ICS syndrome event. MV initiated before the index event was not included in this analysis because no post-index syndrome status could be assigned to that event. Re-initiation after extubation was not counted as a new event, and each eligible MV initiation was assigned to the current ICS syndrome status at the documented time of initiation. The primary outcome was ICU death during the first ICU admission.

The first documented episode of the biomarker-defined ICS syndrome was used as the index event. After this event, each patient’s ICU course was classified according to the current syndrome status. ICS syndrome present was defined by concurrent positivity of all three current biomarker criteria, whereas ICS syndrome absent was defined by failure of at least one current criterion to meet its threshold. Absence of the syndrome therefore indicated only non-fulfillment of the operational laboratory definition and did not imply clinical recovery, resolution of CCI, or absence of a possible contribution from preceding syndrome-positive periods to subsequent outcomes. The index event was an operational starting point for longitudinal observation and was considered the onset of CCI.

The temporal resolution was one calendar day. At the index event, all three biomarkers were required within the same 24 h window. Thereafter, the most recent value of each biomarker was carried forward until the next measurement. No maximum carry-forward duration was imposed; the most recent available value remained active until it was replaced by a new measurement. When multiple results were available on the same day, the highest CRP and the lowest albumin and lymphocyte values were selected. Each post-index day was classified as ICS syndrome present if all three current values met their thresholds and as ICS syndrome absent if at least one did not. A transition occurred when a newly available result changed the syndrome status; events occurring on that calendar day were assigned to the resulting daily syndrome status. The carry-forward rule was part of construction of the time-dependent exposure and was distinct from statistical imputation of model covariates or outcomes.

Data extraction and initial database queries were performed using DB Browser for SQLite version 3.13.1.

### 2.4. Statistical Analysis

Continuous variables are presented as median [IQR], and categorical variables are presented as absolute numbers and percentages. No model-based imputation of missing covariates or outcomes was performed. Analyses used available data, with variable-specific denominators reported in Table 1. For descriptive summaries, repeated quantitative measurements were summarized as patient-level, syndrome-status-specific mean values. Binary variables were summarized as the number and percentage of patients in whom the event occurred during the specified syndrome-status category. A patient could contribute observations to both categories.

The difference between the syndrome-status denominators for MV initiation and those for SOFA, SOFA-2, and selected laboratory variables reflects variable availability rather than exclusion of additional patients. MV initiation could be ascertained from the intervention record for every patient contributing to a syndrome-status category, whereas a score or laboratory summary required at least one valid contributing measurement within that category. In particular, total SOFA and SOFA-2 scores were reported only when the required component data were available; missing components were not treated as normal.

The primary comparison of period-specific clinical, laboratory, and life-support variables was performed between periods with ICS syndrome present and absent. For quantitative variables, between-status differences were estimated using linear mixed-effects models with current syndrome status as a fixed effect and a random intercept for each hospitalization identifier. The effect estimate was reported as the model-based mean difference for ICS syndrome present versus absent with the corresponding 95% CI; positive values indicated higher values while the syndrome was present. For binary outcomes, ORs for ICS syndrome present versus absent were estimated using logistic generalized estimating equation models with an exchangeable working correlation structure to account for repeated observations within hospitalization identifiers. OR > 1 indicated higher odds while the syndrome was present. *P*-values for multiple comparisons were adjusted using the Benjamini−Hochberg FDR procedure.

Mortality density was calculated as the number of ICU deaths per 1000 patient-days assigned to periods with the ICS syndrome present and absent. The between-status difference in mortality density was summarized using the incidence rate ratio (IRR) with a 95% CI.

To evaluate the association between the current presence versus absence of the biomarker-defined ICS syndrome and ICU death, a time-dependent survival analysis was performed. Follow-up started at the index ICS syndrome event and continued until ICU death during the first ICU admission or ICU discharge, with discharge treated as a competing event terminating further risk of ICU death. Accordingly, the Cox model estimated the cause-specific hazard of ICU death among patients remaining in the ICU. The longitudinal dataset was restructured into interval-level records reflecting changes in current syndrome status over time, and patients could sequentially transition between periods with the syndrome present and absent. This model estimates the instantaneous hazard of death while the ICS syndrome is currently present compared with when it is absent; it does not estimate the effect of cumulative syndrome-positive exposure accrued before a given time.

The primary survival analysis used a Cox proportional hazards model with current syndrome status as a time-dependent exposure. The model was fitted in start–stop format, with multiple observation intervals linked to the same patient. Huber–White sandwich robust standard errors clustered at the patient level were used to account for within-patient dependence, and tied event times were handled using the Efron method. The effect estimate was reported as the HR for ICU death for current presence versus absence of the biomarker-defined ICS syndrome, with a 95% CI. An unadjusted model and a minimally adjusted model including sex, age, and BMI were fitted. Complete-case analysis for these covariates included all patients. The adjusted estimate should be interpreted as an association conditional on these three baseline variables, not as a fully adjusted causal or independent effect.

Among patients who died during follow-up, the time from the start of the current syndrome-status interval to ICU death was calculated separately for deaths occurring with the ICS syndrome present and absent. Simon-Makuch survival curves and cumulative hazard curves were constructed to visualize the association with current time-dependent syndrome status. These curves describe mortality according to the syndrome status occupied over follow-up and were not interpreted as a dose-response analysis of cumulative prior time with the syndrome present.

All tests were two-sided, and *p* < 0.05 was considered statistically significant. Descriptive statistics were performed using IBM SPSS Statistics, Version 27.0 (IBM Corp., Armonk, NY, USA). Linear mixed-effects models, generalized estimating equation models, time-dependent Cox regression models, Simon–Makuch survival curves, cumulative hazard curves, and related visualizations were constructed in Python version 3.11.5 using pandas and NumPy for data restructuring, statsmodels for mixed-effects and generalized estimating equation models, lifelines for survival analysis, and matplotlib for visualization.

## 3. Results

### 3.1. General Characteristics of the Cohort

The time-dependent syndrome-status analysis included 820 patients. After the index ICS syndrome event, the total classified follow-up was 23,796 patient-days, of which 5271 patient-days corresponded to periods with ICS syndrome present and 18,525 patient-days corresponded to periods with ICS syndrome absent (Figure 1).

Men accounted for 52.9% of the cohort (434/820), the median age was 68 [55; 77] years, and the median BMI was 25.1 [22.1; 29.3] kg/m^2^. The most common admission conditions were pneumonia in 474 patients (57.8%), ischemic stroke in 403 patients (49.1%), hemorrhagic stroke in 153 patients (18.7%), and traumatic brain injury in 114 patients (13.9%). Diagnostic categories were not mutually exclusive; therefore, their percentages sum to more than 100%. At ICU admission, the median GCS score was 10 [8; 13], the median FOUR score was 13 [10; 15], and the median SOFA-2 score was 3 [2; 5]. The median interval between measurements was 3 days (IQR: 2–4 days) for CRP, 3 days (IQR: 2–4 days) for albumin, and 3 days (IQR: 1–4 days) for lymphocyte count.

Periods with ICS syndrome present were observed in all 820 patients, whereas 700 patients (85.4%) also entered at least one period with ICS syndrome absent; the remaining 120 patients (14.6%) remained continuously syndrome-positive during ICU stay. Overall, 2366 observation intervals were defined, including 1300 intervals with ICS syndrome present and 1066 with ICS syndrome absent. During the first ICU admission, 243 deaths occurred: 133 (54.7%) while the ICS syndrome was present and 110 (45.3%) while it was absent. Despite the shorter cumulative observation time, mortality density was 25.23 deaths per 1000 patient-days while the ICS syndrome was present versus 5.94 per 1000 patient-days while it was absent.

### 3.2. Comparison of Periods with ICS Syndrome Present and Absent

Among variables not used to define syndrome status, periods with ICS syndrome present were associated with higher SOFA and SOFA-2 scores (*p* < 0.001, Table 1), with the largest differences in respiratory, coagulation, and CNS domains. GCS and FOUR scores were lower (both *p* < 0.001), neutrophil counts were higher, and platelet and total protein levels were lower while the ICS syndrome was present (all *p* < 0.001). First MV initiation was also more frequent while the ICS syndrome was present (32.8% vs. 23.6%; *p* < 0.001). As expected by construction, periods with the ICS syndrome present had higher CRP and lower albumin and lymphocyte counts; these defining biomarkers are reported separately in Table 1 and are not interpreted as external evidence of physiological distinctiveness.

The current presence of the biomarker-defined ICS syndrome was associated with a higher hazard of ICU death than its absence (unadjusted HR: 4.02, 95% CI: 2.14–7.55; *p* < 0.001). The association remained after adjustment for sex, age, and BMI (HR: 3.86, 95% CI: 1.86–8.02; *p* < 0.001; Table 2).

Simon-Makuch curves showed a more rapid decline in survival while the ICS syndrome was present than while it was absent (Figure 2). This visualization concerns current time-dependent syndrome status and does not quantify the effect of cumulative prior exposure.

Among patients who died during post-index follow-up, the median time from the start of the current syndrome-status interval to ICU death was 4 [2; 7] days for deaths occurring with the ICS syndrome present and 4 [1; 15] days for deaths occurring with the syndrome absent.

The cumulative hazard also increased more rapidly during follow-up time with the ICS syndrome present, indicating that ICU deaths occurred preferentially while the syndrome was currently present (Figure 3).

## 4. Discussion

### 4.1. Key Findings

In this cohort of 820 patients selected by an index biomarker-defined ICS syndrome event, periods with the syndrome present accounted for 22.2% of classified post-index patient-days but 54.7% of ICU deaths. Variables external to the syndrome definition indicated greater contemporaneous severity while the ICS syndrome was present: organ dysfunction scores were higher (higher SOFA and SOFA-2 scores, *p* < 0.001), neurological scores were lower (GCS and FOUR scores, *p* < 0.001), platelet and total protein levels were lower, and first MV initiation was more frequent (32.8% vs. 23.6%; OR: 1.59, *p* < 0.001). Mortality density was more than four times higher while the syndrome was present (25.23 vs. 5.94 per 1000 patient-days; IRR: 4.25, 95% CI: 3.30–5.47). The time-dependent Cox model estimated a higher instantaneous hazard of ICU death while the ICS syndrome was currently present than while it was absent (unadjusted HR: 4.02, 95% CI: 2.14–7.55; adjusted HR: 3.86, 95% CI: 1.86–8.02). These findings establish a strong current-status association but do not test whether cumulative prior time with the syndrome present independently determines survival.

### 4.2. Relationship with Previous Studies

Our study both confirms and extends the existing literature on chronic critical illness and the persistent inflammation, catabolism, and immunosuppression triad. Nakamura and colleagues established optimal biomarker cut-offs (CRP > 2.0 mg/dL; albumin < 3.0 g/dL; lymphocyte count < 800/μL) using machine learning and single time-point measurements [12]. While we adopted the same thresholds, we move beyond static assessments by examining the temporal dynamics of all three components throughout the ICU course, addressing the acknowledged gap that the underlying biological pattern is complex and not necessarily stable. Therefore, the present study extends previous static and subphenotype-based approaches through longitudinal assessment of the co-occurrence of inflammation, catabolism, and immunosuppression throughout the ICU course and demonstrates that the biomarker-defined ICS syndrome may alternate between present and absent rather than remain fixed.

Our findings also complement the cross-sectional subphenotyping work of Liu and colleagues, who identified four CCI phenotypes based on day-14 characteristics [13]. Patients in our cohort moved between periods with the biomarker-defined ICS syndrome present and absent, suggesting that longitudinal biological classifications may not remain stable throughout an ICU stay. This perspective is also consistent with Darvall and colleagues’ observation that acquired complications commonly contribute to CCI [14]. However, our observational data cannot determine whether the presence of the ICS syndrome precedes, causes or simply coincides with clinical decompensation.

Epidemiological studies from Turkey, Spain, and Tunis reported widely varying CCI prevalence and mortality depending on definitions and populations [15,16]. Our work focuses on a cohort selected by an index biomarker-defined ICS syndrome event and adds a longitudinal comparison according to current syndrome status. Time-dependent survival analysis showed that the current presence of the ICS syndrome was associated with an approximately four-fold higher hazard of ICU death than its absence.

Collectively, these observations provide evidence that concurrent positivity of all three ICS biomarkers identifies periods in which the biomarker-defined ICS syndrome is present and contemporaneous mortality risk is substantially elevated. They do not establish a causal effect of syndrome presence or a dose-response effect of cumulative syndrome-positive duration.

### 4.3. Significance of Study Findings

These observations inform interpretation of biomarker-defined ICS syndrome status and generate hypotheses for future studies of chronic critical illness; their direct implications for management require prospective evaluation.

First, the presence of the ICS syndrome coincided with greater clinical severity. The differences in CRP, albumin, and lymphocyte count are tautological because these variables define syndrome presence and therefore cannot validate it. More informative findings are the differences in variables outside the definition, including SOFA and SOFA-2, GCS, FOUR, platelet count, total protein, and first MV initiation. Notably, the organ-domain differences were not uniform: the largest differences involved the respiratory, coagulation, and CNS domains, whereas cardiovascular and renal domains did not differ significantly. This pattern argues against a simple parallel worsening of all organ systems, although it does not establish a distinct pathophysiological phenotype. Even these associations do not prove that the biomarker-defined ICS syndrome is a distinct causal pathophysiological entity: the three biomarkers may instead function as a composite marker of contemporaneous illness severity.

Second, the survival analysis addresses current syndrome status, not cumulative exposure or an earlier prognostic state. The HR compares the instantaneous hazard of death while the ICS syndrome is currently present with that while it is absent. Neither the Cox model nor the Simon-Makuch curves demonstrate that each additional prior day with the syndrome present incrementally increases later risk. Such a claim would require a separate model of cumulative syndrome-positive days or the proportion of follow-up with the syndrome present. The median interval from the start of the current syndrome-status interval to death was four days in both groups, leaving open the possibility that syndrome presence may partly capture the biochemical signature of terminal deterioration rather than a preceding modifiable process.

Third, the first biomarker-defined ICS syndrome event was used as an operational index for this analysis, not as a proposed universal definition of CCI onset. The observed association supports closer monitoring and further clinical evaluation when the ICS syndrome is present, but it does not show that treatment escalation or shortening periods with the syndrome present improves outcome. Importantly, periods with the ICS syndrome absent should not be interpreted as clinical recovery or resolution of CCI. They may still offer opportunities for rehabilitation and weaning, but this hypothesis requires prospective evaluation.

### 4.4. Strengths and Limitations

The principal strength of this study is its time-dependent design, which preserves changes in current biomarker-defined ICS syndrome status rather than assigning each patient permanently to a single group. Simon-Makuch and cumulative hazard curves complement the interval-level Cox model, and the large real-world cohort provides substantial observation time. The interpretation nevertheless remains associative rather than causal.

Several limitations should be emphasized. Laboratory testing was determined by clinical need rather than a protocol. No maximum carry-forward duration was imposed, and older biomarker values could therefore remain active during less frequently tested periods, potentially resulting in syndrome-status misclassification. Deteriorating patients, particularly those approaching death, may have been tested more frequently, allowing more accurate identification of current ICS syndrome status. This informative observation process could inflate the observed association with mortality; a fixed 24–48 h lag would assess preceding rather than current syndrome status and would not eliminate bias related to clinician-directed testing. The incomplete availability of SOFA, GCS, and FOUR measurements may also have introduced selective missingness, particularly if neurological scores were less frequently documented during deep sedation or severe neurological impairment; the direction and magnitude of any resulting bias cannot be determined from the available data. The model was adjusted only for sex, age, and BMI; residual confounding by case mix, baseline organ dysfunction, neurological injury, organ support, comorbidity, and treatment limitations remains likely, and the estimate should not be called independent. Contemporaneous SOFA, neurological scores, MV, and vasoactive support may also lie on the causal or measurement pathway and were therefore treated as correlates rather than adjustment variables. The proportionality of the syndrome-status effect beyond the specified time-dependent switching was not examined in an additional diagnostic analysis. ICU discharge is related to clinical recovery and current illness severity; accordingly, the reported HR should be interpreted as the cause-specific hazard of ICU death during ongoing ICU stay, rather than as an effect on the cumulative incidence of death accounting for discharge. The study did not model cumulative syndrome-positive exposure or transition-specific hazards, and the short interval from syndrome-status transition to death leaves open reverse causality and terminal-phase classification. Finally, the biomarker thresholds and use of the first ICS syndrome event as an operational index are not universally accepted definitions of CCI; external prospective validation is required.

### 4.5. Future Prospects

The relationship between the presence of the biomarker-defined ICS syndrome and clinical deterioration warrants further investigation. Syndrome presence may precede and contribute to decompensation, may reflect contemporaneous complications, or may partly represent a terminal biochemical phenotype. Prospective studies with protocolized biomarker sampling and explicit temporal sequencing of nosocomial pneumonia, sepsis, acute kidney injury, treatment changes, and limitation-of-care decisions are needed to distinguish these possibilities.

Strategies to predict or abbreviate periods with the ICS syndrome present may be worth exploring, but the present study does not demonstrate that reducing the frequency or duration of syndrome-positive periods improves survival. Prospective studies should determine whether protocolized monitoring of CRP, albumin, and lymphocyte count can reliably identify transitions in syndrome status and whether any biomarker-guided intervention changes patient-centered outcomes.

Third, interventional studies should evaluate whether targeted therapies administered during periods in which the ICS syndrome is absent can lower the risk of transition to syndrome presence. Potential approaches include optimization of nutritional support to counteract catabolism, infection prevention measures to mitigate inflammatory triggers, and immunomodulatory therapies to restore immune competence. Randomized controlled trials would be required to establish the efficacy and safety of such strategies.

Finally, generalizability should be assessed in ICU populations with different case mixes and treatment protocols. Multicenter prospective studies could standardize laboratory schedules and reduce informative observation bias. A dedicated multistate analysis of transitions among ICS syndrome present, ICS syndrome absent, ICU death, and discharge would be valuable for estimating transition-specific hazards and the prognostic relevance of recurrent syndrome-positive episodes; this broader analysis is a separate objective for future work.

## 5. Conclusions

In a cohort selected by an index biomarker-defined ICS syndrome event, the current presence of the ICS syndrome was associated with greater contemporaneous clinical severity and an approximately four-fold higher hazard of ICU death than its absence. The findings support dynamic risk assessment, closer monitoring, and further clinical evaluation, although the biomarker combination may partly reflect contemporaneous illness severity and does not establish that the syndrome itself is causal, that cumulative time with the syndrome present independently determines survival, or that more aggressive treatment improves outcome.

## Figures and Tables

**Figure 1:**
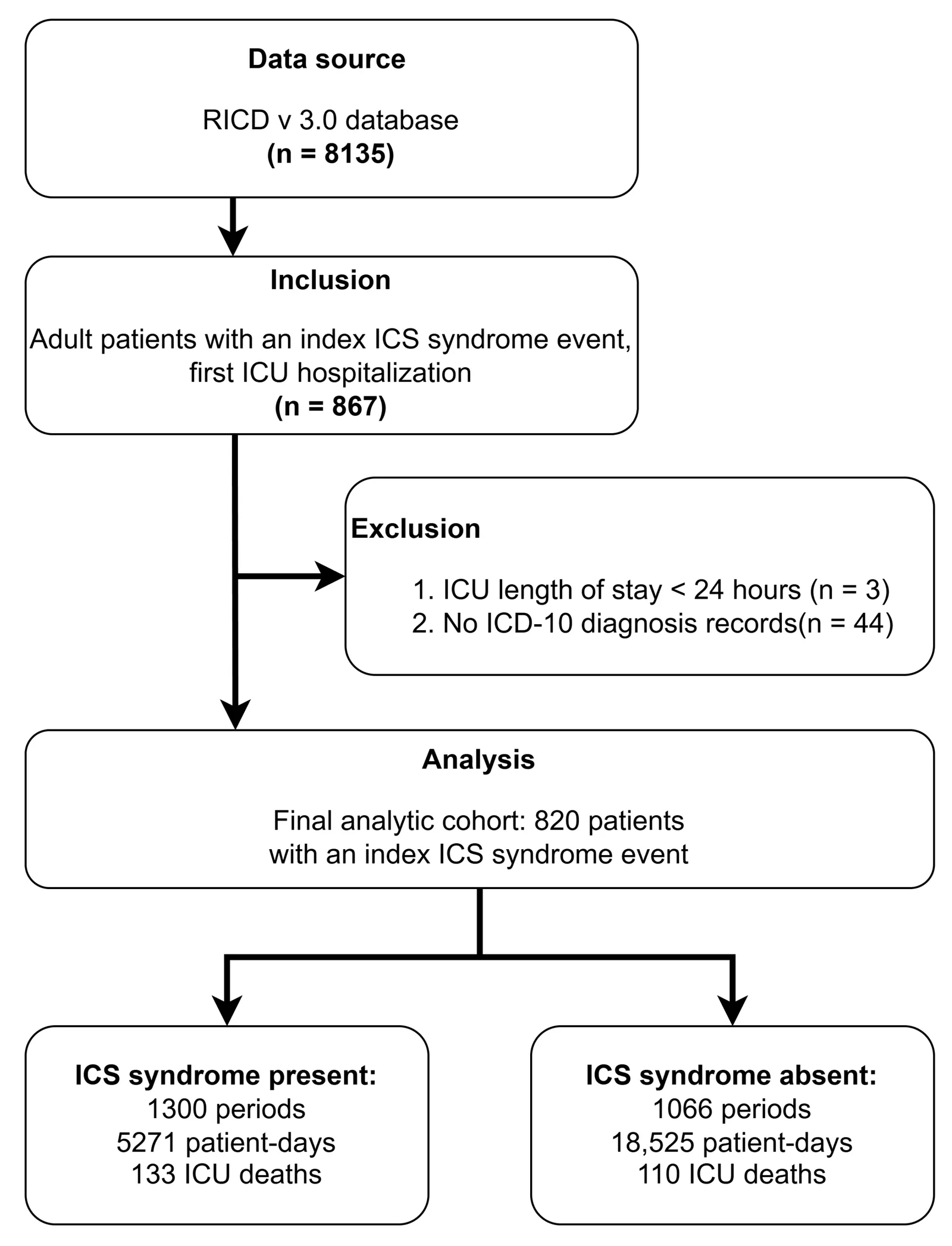
Flowchart of patient selection and inclusion in the time-dependent biomarker-defined ICS syndrome status analysis.

**Figure 2:**
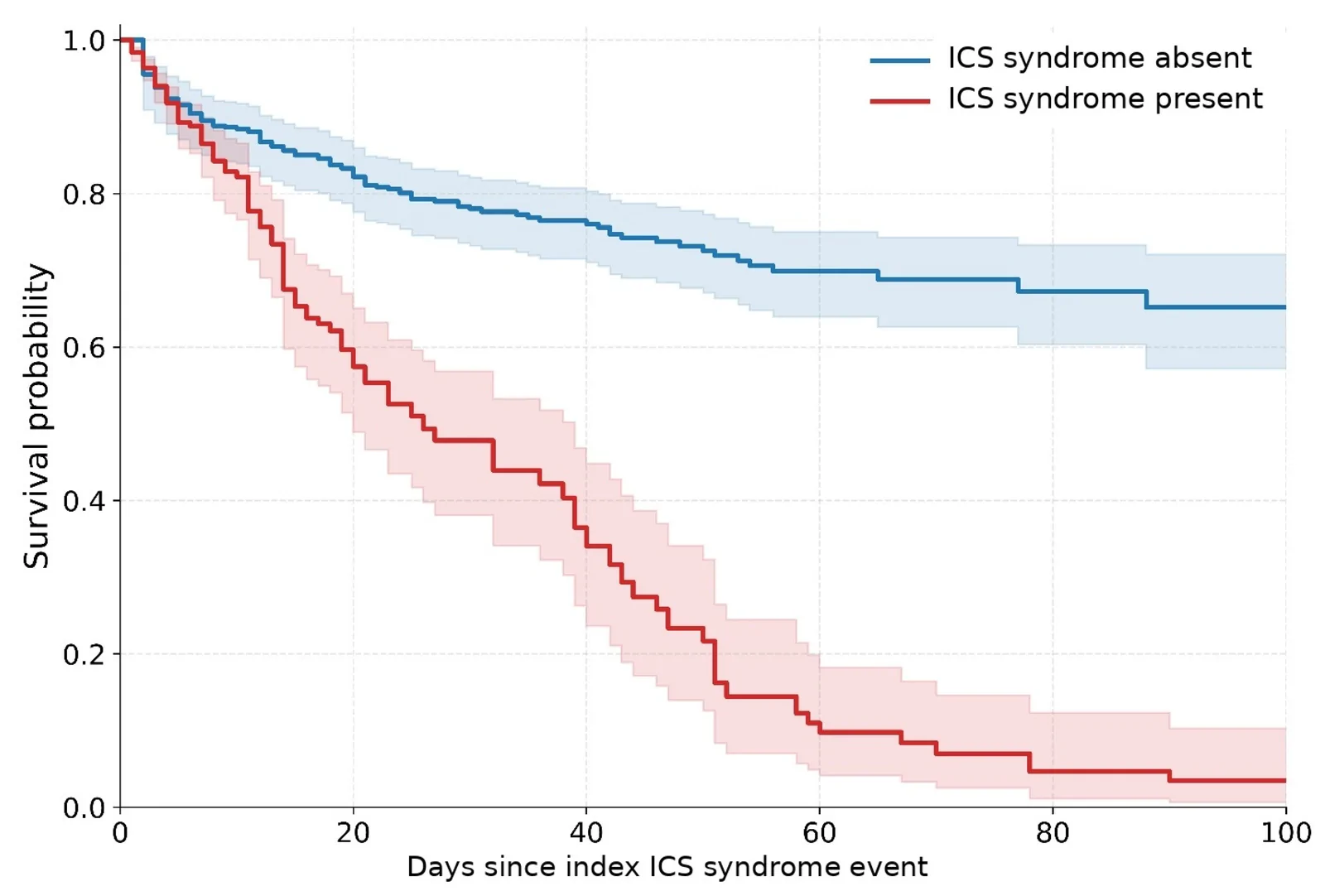
Simon-Makuch survival curves for ICU death according to the current time-dependent presence versus absence of the biomarker-defined ICS syndrome. Abbreviations: ICS, inflammation, catabolism, and immunosuppression syndrome; ICU, intensive care unit.

**Figure 3:**
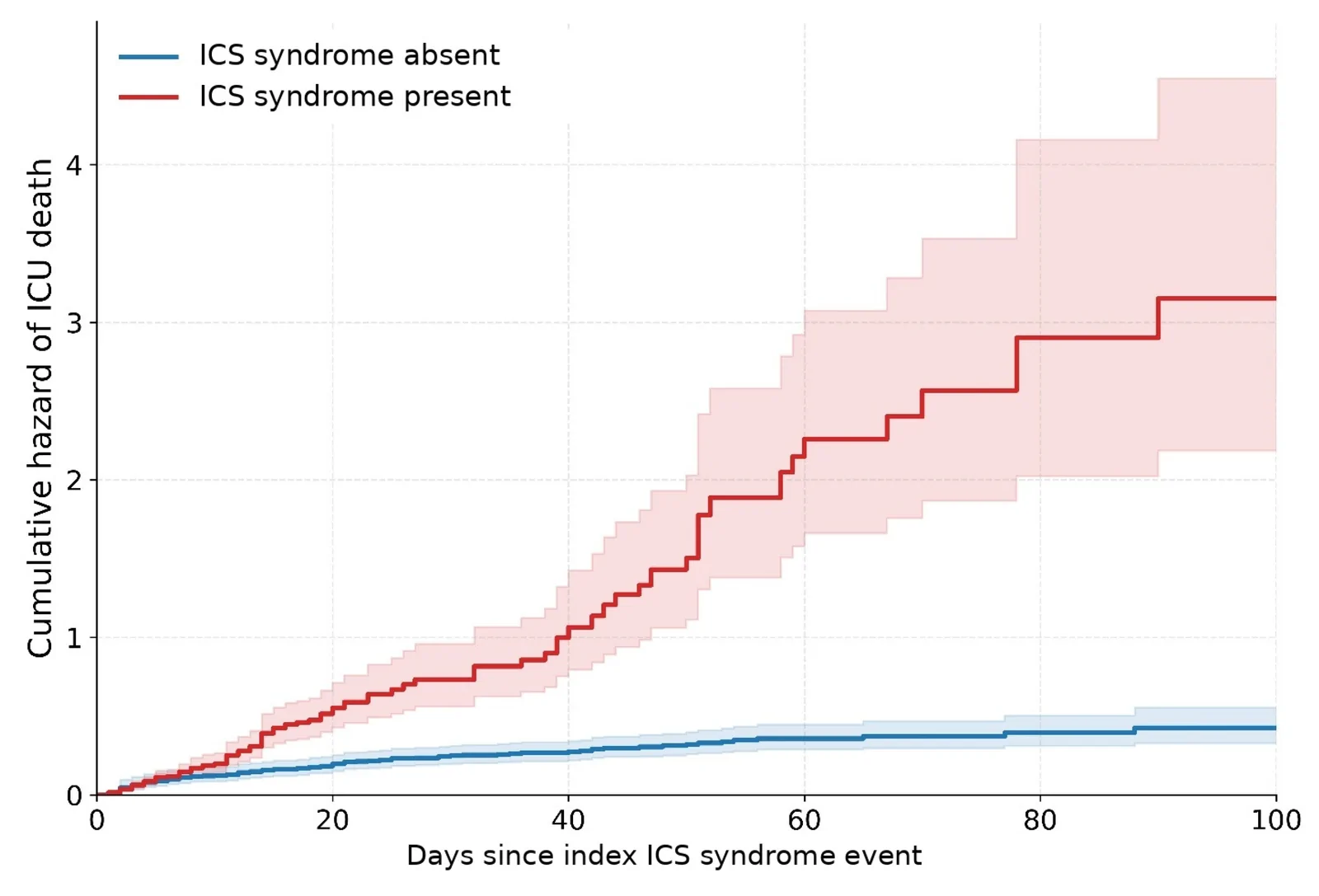
Cumulative hazard of ICU death according to the current presence versus absence of the biomarker-defined ICS syndrome. Abbreviations: ICS, inflammation, catabolism, and immunosuppression; ICU, intensive care unit.

**Table 1:** Comparison of organ dysfunction, laboratory parameters, and life-support variables between periods with ICS syndrome present and absent.

Parameter	ICS Syndrome Present	ICS Syndrome Absent	Difference/OR (95% CI)	FDR-Adjusted *p*-Value
Mean SOFA score, points	n = 531, 5 [3.5; 7.75]	n = 487, 4.25 [3; 6.33]	0.495 [0.258; 0.731]	<0.001
Mean SOFA respiratory domain score, points	n = 531, 1.2 [1; 2]	n = 487, 1.02 [0.91; 1.62]	0.144 [0.061; 0.226]	0.002
Mean SOFA coagulation domain score, points	n = 531, 0 [0; 1]	n = 487, 0 [0; 0.4]	0.133 [0.068; 0.197]	<0.001
Mean SOFA liver domain score, points	n = 530, 0 [0; 0]	n = 487, 0 [0; 0]	0.089 [0.048; 0.130]	<0.001
Mean SOFA cardiovascular domain score, points	n = 531, 0.12 [0; 1.5]	n = 487, 0.25 [0; 1]	−0.025 [−0.131; 0.080]	0.755
Mean SOFA CNS domain score, points	n = 531, 2 [1.2; 3]	n = 487, 2 [1; 3]	0.167 [0.095; 0.239]	<0.001
Mean SOFA renal domain score, points	n = 531, 0 [0; 1]	n = 487, 0 [0; 0.52]	0.047 [−0.011; 0.105]	0.183
Mean SOFA-2 score, points	n = 520, 4 [2.5; 6]	n = 480, 3 [2; 4.21]	0.653 [0.444; 0.862]	<0.001
Mean SOFA-2 respiratory domain score, points	n = 531, 0.5 [0; 1]	n = 487, 0.29 [0; 0.84]	0.164 [0.093; 0.234]	<0.001
Mean SOFA-2 coagulation domain score, points	n = 531, 0 [0; 1]	n = 487, 0 [0; 0.41]	0.161 [0.080; 0.242]	<0.001
Mean SOFA-2 liver domain score, points	n = 531, 0 [0; 0]	n = 487, 0 [0; 0]	0.080 [0.046; 0.115]	<0.001
Mean SOFA-2 cardiovascular domain score, points	n = 520, 0 [0; 0.5]	n = 480, 0.05 [0; 0.39]	−0.008 [−0.056; 0.039]	0.824
Mean SOFA-2 CNS domain score, points	n = 531, 2 [1.17; 3]	n = 487, 2 [1; 2.35]	0.184 [0.114; 0.253]	<0.001
Mean SOFA-2 renal domain score, points	n = 531, 0 [0; 1]	n = 487, 0 [0; 0.57]	0.080 [0.006; 0.153]	0.067
Mean GCS score, points	n = 367, 10 [7.25; 12]	n = 417, 10.33 [8; 12.5]	−0.627 [−0.906; −0.348]	<0.001
Mean FOUR score, points	n = 356, 12 [9; 14]	n = 412, 12.58 [10; 14.5]	−0.879 [−1.235; −0.522]	<0.001
Mean creatinine, µmol/L	n = 817, 81.5 [60.3; 125.63]	n = 695, 74.18 [56; 113.17]	−0.347 [−3.518; 2.825]	0.897
Mean urea, mmol/L	n = 809, 7.71 [5.1; 13.8]	n = 685, 6.85 [4.5; 11.74]	0.329 [−0.080; 0.738]	0.189
Mean arterial lactate, mmol/L	n = 284, 1.5 [1.1; 2.5]	n = 261, 1.35 [1; 2.1]	0.139 [−0.190; 0.467]	0.546
Mean hemoglobin, g/L	n = 820, 92.4 [84.29; 104.33]	n = 700, 92.44 [85.63; 102.25]	0.229 [−0.790; 1.248]	0.770
Mean WBC count, ×10^9^/L	n = 820, 9.2 [6.4; 12.83]	n = 700, 8.89 [7.26; 11.93]	0.007 [−0.400; 0.414]	0.995
Mean neutrophil count, ×10^9^/L	n = 820, 7.98 [5.21; 11.44]	n = 700, 6.81 [5.12; 9.7]	0.965 [0.581; 1.348]	<0.001
Mean platelet count, ×10^9^/L	n = 820, 204 [142.15; 270.71]	n = 700, 246.89 [179.57; 315.53]	−32.679 [−38.707; −26.651]	<0.001
Mean AST, U/L	n = 816, 34.45 [22.68; 54.86]	n = 693, 30.46 [20.87; 48.73]	0.053 [−24.271; 24.376]	0.997
Mean ALT, U/L	n = 769, 28.8 [17.45; 55.7]	n = 664, 27.5 [15.5; 49.99]	−0.132 [−11.090; 10.825]	0.995
Mean ALP, U/L	n = 641, 98.65 [73.1; 150.4]	n = 584, 103.51 [77.67; 148.43]	−2.063 [−7.586; 3.460]	0.597
Mean total protein, g/L	n = 820, 50.12 [46.35; 54.33]	n = 693, 52.23 [48.35; 56.59]	−1.704 [−2.081; −1.328]	<0.001
Mean albumin, g/L	n = 820, 24.4 [22; 26.63]	n = 679, 25.2 [22.44; 28.1]	−0.891 [−1.131; −0.652]	<0.001
Mean CRP, mg/L	n = 820, 110.16 [62.91; 175.88]	n = 690, 74.29 [44.04; 128.83]	28.119 [23.186; 33.052]	<0.001
Mean lymphocyte count, ×10^9^/L	n = 820, 0.6 [0.5; 0.7]	n = 700, 1.24 [1; 1.62]	−0.755 [−0.807; −0.703]	<0.001
MV initiation, n (%)	n = 820; n = 269 (32.8%)	n = 700; n = 165 (23.6%)	1.594 [1.302; 1.952]	<0.001

Notes: CRP, albumin, and lymphocyte count are defining biomarkers; their between-status differences are expected by construction and should not be interpreted as external validation. All other variables are non-defining correlates. Quantitative variables represent patient-level mean values calculated within each syndrome-status category. N is the number of patients contributing at least one valid observation for the variable in that category. MV initiation was ascertainable for all patients contributing to each category, explaining denominators of 820 and 700. ALP, alkaline phosphatase; ALT, alanine aminotransferase; AST, aspartate aminotransferase.

**Table 2:** Mortality density and time-dependent Cox regression for ICU death according to the current presence versus absence of the biomarker-defined ICS syndrome.

Parameter	Estimate [95% CI]	*p*-Value
Incidence rate ratio, ICS syndrome present vs. absent	IRR: 4.25 [3.30–5.47]	<0.001
Unadjusted time-dependent Cox model, the current presence vs. absence of biomarker-defined ICS syndrome	HR: 4.02 [2.14–7.55]	<0.001
Adjusted time-dependent Cox model, the current presence vs. absence of biomarker-defined ICS syndrome (sex, age, BMI; N = 820; deaths = 243)	HR: 3.86 [1.86–8.02]	<0.001

Abbreviations: CI, confidence interval; HR, hazard ratio; ICS, inflammation, catabolism, and immunosuppression; ICU, intensive care unit; IRR, incidence rate ratio.

## Data Availability

Publicly and partially available datasets were analyzed in this study. The RICD dataset can be obtained upon request at https://fnkcrr-database.ru/ (accessed on 1 July 2026).

## Supplementary Materials

The following supporting information can be downloaded at: https://www.mdpi.com/article/10.3390/diagnostics16193254/s1, Supplementary S1. STROBE Statement checklist.

## Abbreviations

The following abbreviations are used in this manuscript:

ALP	alkaline phosphatase
ALT	alanine aminotransferase
AST	aspartate aminotransferase
BMI	body mass index
CCI	chronic critical illness
CI	confidence interval
CNS	central nervous system
CRP	C-reactive protein
FDR	false discovery rate
FOUR	Full Outline of UnResponsiveness
FRCC ICMR	Federal Research and Clinical Center of Intensive Care Medicine and Rehabilitology
GCS	Glasgow Coma Scale
HR	hazard ratio
ICD-10	International Classification of Diseases, 10th Revision
ICU	intensive care unit
ICS syndrome	inflammation, catabolism, and immunosuppression syndrome
IQR	interquartile range
IRR	incidence rate ratio
MV	mechanical ventilation
OR	odds ratio
PICS	persistent inflammation, catabolism, and immunosuppression syndrome
RICD	Russian Intensive Care Dataset
SOFA	Sequential Organ Failure Assessment
SOFA-2	Updated Sequential Organ Failure Assessment
STROBE	Strengthening the Reporting of Observational Studies in Epidemiology
VIS	vasoactive-inotropic score
WBC	white blood cell
