# Supplementary material for: The Critical Clock: Time-Dependent Inflammation, Catabolism and Immunosuppression Syndrome Is Associated with ICU Mortality
Source: Diagnostics (Basel). 2026 Oct 8;16(19):3254. doi: 10.3390/diagnostics16193254 (PMC13664344; doi:10.3390/diagnostics16193254)
Supplement: Supplementary file 1 [file diagnostics-16-03254-s001.zip › diagnostics-4569366-supplementary.pdf]

Supplementary Information

The Critical Clock: Time-Dependent Inflammation, Catabolism and Immunosuppression Syndrome is associated with ICU mortality

Valery Likhvantsev [1,2,*], Levan Berikashvili [1], Petr Polyakov [1], Mikhail Yadgarov [1], Artem Kuzovlev [1], Alexey Yakovlev [1] and Andrey Grechko [1]

[1] Federal Research and Clinical Center of Intensive Care Medicine and Rehabilitology, Moscow, Russia
[2] I.M. Sechenov First Moscow State Medical University of the Russian Ministry of Health, Moscow, Russia
* Correspondence: lik0704@gmail.com

Supplementary S1. STROBE Statement–Checklist.

	Item No	Recommendation	Page No. (Section(s))
**Title and abstract**	1	(*a*) Indicate the study's design with a commonly used term in the title or the abstract	Abstract – Methods
		(*b*) Provide in the abstract an informative and balanced summary of what was done and what was found	Abstract – Background/Objectives; Methods; Results; Conclusions
**Introduction**			
Background/rationale	2	Explain the scientific background and rationale for the investigation being reported	1. Introduction
Objectives	3	State specific objectives, including any prespecified hypotheses	1. Introduction – final paragraph beginning with "Accordingly, the objective was…"
**Methods**			
Study design	4	Present key elements of study design early in the paper	Abstract – Methods; 2.2. Study design and setting
Setting	5	Describe the setting, locations, and relevant dates, including periods of recruitment, exposure, follow-up, and data collection	2.1. Source of data; 2.2. Study design and setting; 2.3. Data management
Participants	6	(*a*) Give the eligibility criteria, and the sources and methods of selection of participants. Describe methods of follow-up	2.1. Source of data; 2.2. Study design and setting; 2.4. Statistical analysis
		(*b*) For matched studies, give matching criteria and number of exposed and unexposed	Not applicable – this was not a matched study
Variables	7	Clearly define all outcomes, exposures, predictors, potential confounders, and effect modifiers. Give diagnostic criteria, if applicable	2.2. Study design and setting; 2.3. Data management; 2.4. Statistical analysis
Data sources/ measurement	8*	For each variable of interest, give sources of data and details of methods of assessment (measurement). Describe comparability of assessment methods if there is more than one group	2.1. Source of data; 2.3. Data management; 2.4. Statistical analysis
Bias	9	Describe any efforts to address potential sources of bias	2.2. Study design and setting; 2.4. Statistical analysis; 4.4. Strengths and limitations

Study size	10	Explain how the study size was arrived at	2.2. Study design and setting – all eligible cases were included without formal sample-size calculation
Quantitative variables	11	Explain how quantitative variables were handled in the analyses. If applicable, describe which groupings were chosen and why	2.3. Data management; 2.4. Statistical analysis; Table 1 – Notes
Statistical methods	12	(*a*) Describe all statistical methods, including those used to control for confounding	2.4. Statistical analysis
		(*b*) Describe any methods used to examine subgroups and interactions	Not applicable – no subgroup or interaction analyses were reported
		(*c*) Explain how missing data were addressed	2.4. Statistical analysis – no imputation; available-case analysis with variable-specific denominators
		(*d*) If applicable, explain how loss to follow-up was addressed	Not applicable – no loss to follow-up occurred; follow-up continued from the index ICS syndrome event until ICU death or ICU discharge
		(*e*) Describe any sensitivity analyses	Not applicable – no sensitivity analyses were performed
**Results**			
Participants	13*	(a) Report numbers of individuals at each stage of study–eg numbers potentially eligible, examined for eligibility, confirmed eligible, included in the study, completing follow-up, and analysed	3.1. General characteristics of the cohort; Figure 1. Flowchart of patient selection and inclusion in the time-dependent biomarker-defined ICS syndrome status analysis
		(b) Give reasons for non-participation at each stage	3.1. General characteristics of the cohort; Figure 1
		(c) Consider use of a flow diagram	Figure 1. Flowchart of patient selection and inclusion in the time-dependent biomarker-defined ICS syndrome status analysis
Descriptive data	14*	(a) Give characteristics of study participants (eg demographic, clinical, social) and information on exposures and potential confounders	3.1. General characteristics of the cohort; Table 1
		(b) Indicate number of participants with missing data for each variable of interest	Table 1; Table 1 – Notes; 2.4. Statistical analysis – variable-specific denominators are reported
		(c) Summarise follow-up time (eg, average and total amount)	3.1. General characteristics of the cohort; Figure 1

Outcome data	15*	Report numbers of outcome events or summary measures over time	Abstract – Results; 3.1. General characteristics of the cohort; 3.2. Comparison of periods with ICS syndrome present and absent; Table 2; Figures 2 and 3
Main results	16	(*a*) Give unadjusted estimates and, if applicable, confounder-adjusted estimates and their precision (eg, 95% confidence interval). Make clear which confounders were adjusted for and why they were included	2.4. Statistical analysis; 3.2. Comparison of periods with ICS syndrome present and absent; Tables 1 and 2
		(*b*) Report category boundaries when continuous variables were categorized	2.2. Study design and setting; 2.3. Data management – CRP >20 mg/L, albumin <30 g/L and lymphocyte count <0.8 × 10^9/L
		(*c*) If relevant, consider translating estimates of relative risk into absolute risk for a meaningful time period	3.1. General characteristics of the cohort; 3.2. Comparison of periods with ICS syndrome present and absent; Table 2 – absolute numbers of deaths and mortality densities per 1,000 patient-days are reported; no additional fixed-time absolute-risk estimate was calculated
Other analyses	17	Report other analyses done–eg analyses of subgroups and interactions, and sensitivity analyses	2.4. Statistical analysis; 3.2. Comparison of periods with ICS syndrome present and absent; Figures 2 and 3 – additional time-dependent survival visualizations and interval-to-death summaries were reported; no subgroup, interaction, or sensitivity analyses were performed
**Discussion**			
Key results	18	Summarise key results with reference to study objectives	4.1. Key findings; 5. Conclusions
Limitations	19	Discuss limitations of the study, taking into account sources of potential bias or imprecision. Discuss both direction and magnitude of any potential bias	4.4. Strengths and limitations
Interpretation	20	Give a cautious overall interpretation of results considering objectives, limitations, multiplicity of analyses, results from similar studies, and other relevant evidence	4.1. Key findings; 4.2. Relationship with previous studies; 4.3. Significance of study findings; 4.4. Strengths and limitations; 5. Conclusions
Generalisability	21	Discuss the generalisability (external validity) of the study results	4.4. Strengths and limitations; 4.5. Future prospects
**Other information**			

Funding	22	Give the source of funding and the role of the funders for the present study and, if applicable, for the original study on which the present article is based	Funding – "This research received no external funding"

*Give information separately for exposed and unexposed groups.

Note: An Explanation and Elaboration article discusses each checklist item and gives methodological background and published examples of transparent reporting. The STROBE checklist is best used in conjunction with this article (freely available on the Web sites of PLoS Medicine at http://www.plosmedicine.org/, Annals of Internal Medicine at http://www.annals.org/, and Epidemiology at http://www.epidem.com/). Information on the STROBE Initiative is available at http://www.strobe-statement.org.
